# Data Retention Characterization of Gate-Injected Gold-Nanoparticle Non-Volatile Memory with Low-Damage CF_4_-Plasma-Treated Blocking Oxide Layer

**DOI:** 10.3390/nano7110385

**Published:** 2017-11-10

**Authors:** Yu-Hua Liu, Chyuan-Haur Kao, Tsung-Chin Cheng, Chih-I Wu, Jer-Chyi Wang

**Affiliations:** 1 Department of Electronic Engineering, Chang Gung University, Guishan Dist., Taoyuan 33302, Taiwan; a22730730@gmail.com (Y.-H.L.); chkao@mail.cgu.edu.tw (C.-H.K.); 2 Department of Electronic Engineering, Ming Chi University of Technology, Taishan Dist., New Taipei City 24301, Taiwan; 3 Kidney Research Center, Department of Nephrology, Chang Gung Memorial Hospital, Linkou, Guishan Dist., Taoyuan 33305, Taiwan; 4 Graduated Institute of Photonics and Optoelectronics, National Taiwan University, Taipei 10617, Taiwan; d99941012@ntu.edu.tw (T.-C.C.); chihiwu@cc.ee.ntu.edu.tw (C.-I.W.); 5 Department of Electrical Engineering, National Taiwan University, Taipei 10617, Taiwan; 6 Electronic and Optoelectronic System Research Laboratories, Industrial Technology Research Institute, Chutung, Hsinchu 31057, Taiwan; 7 Department of Neurosurgery, Chang Gung Memorial Hospital, Linkou, Guishan Dist., Taoyuan 33305, Taiwan

**Keywords:** gold nanoparticle (Au-NP), non-volatile memory (NVM), CF_4_ plasma, blocking oxide, bandgap engineering, gate injection, data retention

## Abstract

Gold-nanoparticle (Au-NP) non-volatile memories (NVMs) with low-damage CF_4_ plasma treatment on the blocking oxide (BO) layer have been investigated to present the gate injection of the holes. These holes, injected from the Al gate with the positive gate bias, were explained by the bandgap engineering of the gradually-fluorinated BO layer and the effective work function modulation of the Al gate. The Si–F complex in the BO layer was analyzed by X-ray photoelectron spectroscopy (XPS), while the depth of fluorine incorporation was verified using a secondary ion mass spectrometer (SIMS). In addition, the valence band modification of the fluorinated BO layer was examined by ultraviolet photoelectron spectroscopy (UPS) to support the bandgap engineering. The reactive power of the CF_4_ plasma treatment on the BO layer was modified to increase the electric field of the BO layer and raise the effective work function of the Al gate, leading to the hole-injection from the gate. The injected holes are trapped at the interface between the gold-nanoparticles (Au-NPs) and the tunneling oxide (TO) layer, resulting in superior data retention properties such as an extremely low charge loss of 5.7% at 10^4^ s and a nearly negligible increase in charge loss at 85 °C of the CF_4_-plasma-treated Au-NP NVMs, which can be applied in highly reliable consumer electronics.

## 1. Introduction

Over the past few years, the demand for portable electronic devices has increased rapidly due to the growth of the Internet of Things (IoT); therefore, a high-density unit is necessary in the development of floating gate (FG), non-volatile memories (NVMs) [1,2]. However, as the devices scale, the charges stored in the FG are easily lost through the thin tunneling oxide (TO) layer, leading to severe reliability issues [3]. To overcome these issues, discrete-charge storage concepts have been introduced in this generation [4,5]. Among these alternative memories, nanocrystal (NC) memories are being extensively investigated. The memories can store charges in isolated nanoparticles (NPs) to suppress lateral migration and enhance data retention behavior [6]. The materials used as NPs can be classified into three major categories: semiconductors, high-*k* dielectrics, and metals. Initially, Si and Ge were used as NPs because of their antecedence in the semiconductor industry [7,8]. Later, Gd_2_O_3_-NC memories were realized, in which crystalline Gd_2_O_3_-NC dots are surrounded by amorphous Gd_2_O_3_ dielectrics to ensure an energy band offset, enabling charge storage [9,10]. To improve the performance of NC memories, metal NPs, which can have a high density of states (DOS) around the Fermi-level, high particle scalability, and a wide range of accessible work functions, have been employed [11]. There are lots of metal NPs, such as Ag, Au, Pt, W, Co, Ni, NiSi_2_, Ni_1−*x*_Fe*_x_*, Hf, TiN, and Al metal NPs, being used for the non-volatile memory (NVM) applications [3]. This approach is to engineer the depth of the potential well (*d*_eff_) at the storage nodes, achieving the asymmetrical barrier between the substrate and the storage nodes for the easy programming and good data-retention properties. For example, NVMs with gold nanoparticles (Au-NPs) have been fabricated with superior reliabilities owing to their large work function, low reactivity, high dot density, and uniform dot distribution [12]. Nevertheless, all NVMs with metal NPs presented the storage of electrons, which were injected from the substrate.

Recently, it has been demonstrated that gate-injected NVMs exhibit superior reliability properties compared to substrate-injected ones because of less operating damage to the TO layer [13]. The bandgap-engineered oxide layers—oxide-nitride-oxide (ONO) stacked layers—on top of the charge-trapping layer have been used to realize the carrier injection from the poly-Si gate. The fabrication of the ultra-thin ONO stacked layers (1.3 nm/2.1 nm/1.7 nm) is too complicated to control their quality and ensure uniform operation in all devices. Previously, NH_3_ plasma treatment has been performed on the TO layer, creating a gradually-nitrided TO layer that forms a built-in electric field and enhances the injection of electrons from the Si substrate [14]. In this work, we propose a novel technique to achieve the Au-NP NVMs with gate-injected holes by using the low-damage CF_4_ plasma treatment on the blocking oxide (BO) layer. The material characterizations of scanning electron microscopy (SEM), high-resolution transmission electron microscopy (HRTEM), X-ray photoelectron spectroscopy (XPS), secondary ion mass spectrometer (SIMS), and ultraviolet photoelectron spectroscopy (UPS) were employed to analyze the film composition, device structure, and energy band diagram of the memory devices. For the CF_4_-plasma-treated Au-NP NVMs, holes were injected from the Al gate with the positive gate bias (*V*_g_), which can be explained by the bandgap engineering of the gradually-fluorinated BO layer and the effective work function modulation of the Al gate. Furthermore, the devices have been found to exhibit superior data retention properties, suitable for highly reliable future NVM applications.

## 2. Materials and Methods

### 2.1. Sample Preparation

**Figure 1:**
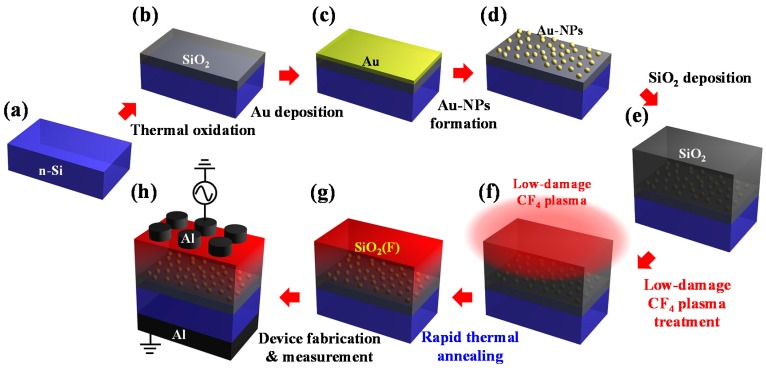
Fabrication procedures of the gold-nanoparticle (Au-NP) non-volatile memories (NVMs) with low-damage CF_4_ plasma treatment on the blocking oxide (BO) layer. (**a**) *n*-type Si (100) wafers with standard Radio Corporation of America (RCA) clean; (**b**) thermally grown SiO_2_ film; (**c**) physical-vapor-deposition (PVD)-deposited Au film; (**d**) rapid thermal annealing (RTA) to form Au-NPs; (**e**) plasma-enhanced chemical vapor deposition (PECVD)-deposited SiO_2_ film; (**f**) low-damage CF_4_ plasma treatment; (**g**) RTA to activate fluorine ions; and (**h**) Al contact formation to fabricate Au-NP NVMs.

The Al/SiO_2_/Au-NPs/SiO_2_/*n*-Si structure with low-damage CF_4_ plasma treatment on the BO layer was fabricated to form the Au-NP NVMs. The *n*-type Si (100) wafers were first cleaned using the standard Radio Corporation of America (RCA) clean. Next, a 6-nm-thick SiO_2_ layer was grown on the wafers in a horizontal furnace at 950 °C for 5 min in an O_2_ gas ambient as the TO layer. Then, a 2-nm-thick Au film was deposited by a thermal evaporator at 10^−6^ Torr with a pure Au bullet (99.99% purity). Subsequently, each sample was subjected to rapid thermal annealing (RTA) at 700 °C for 30 s to form Au-NPs. After the Au-NPs had formed, a 20-nm-thick SiO_2_ layer was deposited via plasma-enhanced chemical vapor deposition (PECVD) as the BO layer, where the samples were kept in a SiH_4_ and N_2_O ambient at a radio frequency (RF) power of 50 W with gas flow rates of 5 and 200 sccm, respectively. Thereafter, a low-damage CF_4_ plasma treatment was performed in a PECVD system with a quartz filter, filtering high-energy electrons, ions, and ultra-violet (UV) radiation to reduce plasma damage on the BO layer [15]. The chamber was first evacuated to 10^−6^ Torr and gradually heated to 300 °C. Then, the CF_4_ gas ambient of 50 sccm was flowed into the chamber to keep the pressure at 500 mTorr. The system at RF powers of 20, 30, and 50 W was performed on the BO layer for 30 s and labeled as 20 W, 30 W, and 50 W, respectively. Additionally, a sample without the treatment was also fabricated for comparison and labeled as w/o. Then, a RTA was performed at 700 °C for 30 s to activate the fluorine ions. Finally, a 300-nm-thick Al film was deposited by a thermal evaporator at 10^−6^ Torr with a pure Al bullet (99.999% purity), and a gate was photo-lithographically defined and then etched. The entire fabrication process of the Au-NP NVMs with low-damage CF_4_ plasma treatment on the BO layer is illustrated in Figure 1.

### 2.2. Characterization

To understand the incorporation of fluorine atoms in the BO layer, its elemental composition was obtained using SIMS (ION-TOF GmbH, Münster, Germany) depth profiling. Moreover, the chemical bonding of the CF_4_-plasma-treated SiO_2_ layer was examined using XPS (Waters Corp., Milford, MA, USA) analysis. The dot size and density of Au-NPs were observed by SEM (JEOL Ltd., Tokyo, Japan). Further, the stacked films of the devices with Au-NPs were confirmed by HRTEM images using a scanning transmission electron microscope (STEM) system (Thermo Fisher Scientific Inc., Hillsboro, OR, USA). Besides, the ionization energy of the BO layer with low-damage CF_4_ plasma treatment was obtained via UPS (RBD Instruments, Inc., Bend, OR, USA). For the electrical characterization of the CF_4_-plasma-treated Au-NP NVMs, the current density versus voltage (*J*-*V*) and high-frequency (1 MHz) capacitance versus voltage (*C*-*V*) curves were measured using Keithley 4200-SCS semiconductor characterization system (Tektronix, Inc., Beaverton, OR, USA), and the gate pulse was supplied by Keithley 4225-PMU ultra-fast IV unit (Tektronix, Inc., Beaverton, OR, USA) to operate the devices.

## 3. Results and Discussion

### 3.1. Material Analysis

**Figure 2:**
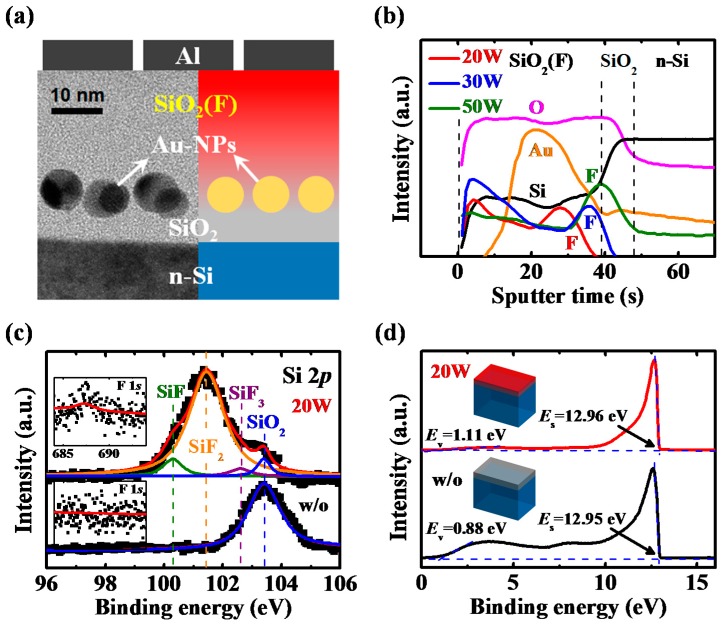
(**a**) Cross-sectional structure and high-resolution transmission electron microscopy (HRTEM) image of the Au-NP NVMs; (**b**) secondary ion mass spectrometer (SIMS) depth profiles of the CF_4_-plasma-treated Au-NP NVMs; (**c**) X-ray photoelectron spectroscopy (XPS) spectra of Si 2*p* and F 1*s* of the BO layer and (**d**) ultraviolet photoelectron spectroscopy (UPS) spectra of the SiO_2_ thin films with and without the treatment at 20 W. The energy from the valence band to vacuum level (*E*_vac_vb_) of the SiO_2_ films with and without the CF_4_ plasma treatment at 20 W was found to be 9.13 and 9.35 eV, respectively.

Figure 2a shows the cross-sectional structure and HRTEM image of the Au-NP NVMs. The HRTEM image with a lower magnification was also presented in Figure S1 of the Supplementary Materials to show more Au-NPs on the SiO_2_ layer. The Au-NPs are formed by a dewetting process of a deposited Au film. There are some literatures illustrating the major driving forces that contribute to the dewetting process of Au-NPs [16,17,18,19,20,21,22]. The process is achieved through the melting of the Au film and the relaxation of the Au film stress on the SiO_2_ layer, which is limited by the surface mobility of Au atoms. Some long-range forces such as the dispersion force and the electrical double layers will also affect the nanoparticle (NP) size and location distributions. To reduce the elastic energy induced by the film stress during the deposition and thermal processes, the Au film tends to break into some islands along the initial film thickness perturbation. The final geometry depends on the balance between these driving forces mentioned above. Thus, the formation of Au-NPs on the SiO_2_ layer is realized and the dot size and density are established. It is reported that the thickness of the deposited Au film and the following rapid thermal process (RTP) can be controlled to modify the Au-NP size and density on the SiO_2_ layer [23,24,25,26] and to present good reliability properties of the Au-NP NVMs [27]. In the work, to obtain suitable memory characteristics, the Au film of 2 nm was deposited and the following RTA of 700 °C for 30 s was performed to achieve the Au-NP density of 1.2 × 10^12^ cm^−2^ and the average Au-NP diameter of approximately 8.4 nm, as shown in the SEM image of Figure S2 of the Supplementary Materials, which is almost the same as that seen in the HRTEM image (Figure 2a). Generally speaking, the specimen for the cross-sectional HRTEM is widely prepared by using the focus ion beam (FIB) because of the ease of obtaining the sample at a specific site with a constant thickness [28]. However, it is difficult to control the amount of remaining nanoparticles for the specimen preparation of the Au-NP NVMs. The different amounts of the remaining Au-NPs at the specimen surface are responsible for the different contrasts of the Au-NPs observed in the cross-sectional HRTEM image. The image also confirmed the film thicknesses of the TO and BO layers for the further study of carrier injection.

Figure 2b presents the SIMS depth profiles of the Au-NP NVMs with different reactive powers of the low-damage CF_4_ plasma treatment on the BO layer. With the increase in reactive power of CF_4_ plasma, more fluorine ions were incorporated in the BO layer, diffusing deep into the film. In general, when the impurities diffuse in a film the impurity concentration would decrease from the surface to the bulk without any fluctuation. However, if there are stacked films, the impurities such as the fluorine ions will pile up at the interface of the films, as proposed by Chang et al. [29]. In Au-NP NVMs, there are lots of Au-NPs embedded within the SiO_2_ film. Thus, the fluorine ions will accumulate at the interface between the Au-NPs and the SiO_2_ film, leading to the intensity fluctuation of fluorine, as shown in Figure 2b. When the CF_4_ plasma power increases to 50 W, more fluorine ions are generated and the position of the maximum fluorine intensity shifts toward the interface between the Au-NPs and the TO layer. To understand the chemical reaction between the CF_4_ plasma and the BO layer, the XPS spectra of Si 2*p* and F 1*s* of the BO layer with and without the treatment at 20 W were analyzed (Figure 2c). The BO layer without treatment depicted a pure SiO_2_ layer with a Si 2*p* spectrum at 103.4 eV. On the other hand, for the BO layer with the CF_4_ plasma treatment at 20 W, three additional spectra at 100.3, 101.45, and 102.6 eV were obtained, indicating the Si–F, Si–F_2_, and Si–F_3_ bonds, respectively [30]. The fluorine-rich chemistry in SiO_2_ film with a complex reaction product can be observed at the F 1*s* spectrum of 687.25 eV for the CF_4_-plasma-treated sample [31]. Figure 2d shows the UPS spectra of the SiO_2_ thin films with and without the low-damage CF_4_ plasma treatment at 20 W. Following sputtering the SiO_2_ film surface to remove the hydrocarbon contamination with Ar^+^ ions at 4 keV for 2 min, UPS was carried out using a UV He lamp (21.2 eV) in an ultra-high vacuum chamber. A broad spectral feature representative of the valence band (*E*_v_) of the SiO_2_ films was observed at a much lower binding energy [32]. Linear extrapolation of the left of the peak curve made it possible to estimate the valence-band maximum (*VBM*) level energy. The photoemission threshold (*E*_s_) was evaluated by a linear fit of the beginning of the right defined by the secondary electron peak. Thus, the energy from the valence band to vacuum level (*E*_vac_vb_) can be calculated by the following equation [33]:

(1) $$E_{\mathrm{vac}\_\mathrm{vb}}=h\nu -(E_{S}-VBM)$$

where *hν* is the incident He. Thus, the *E*_vac_vb_ of the SiO_2_ films with and without the CF_4_ plasma treatment was determined to be 9.13 and 9.35 eV, respectively, indicating a light increase in the *E*_vac_vb_ of the SiO_2_ films with the treatment.

### 3.2. Electrical Characteristics

**Figure 3:**
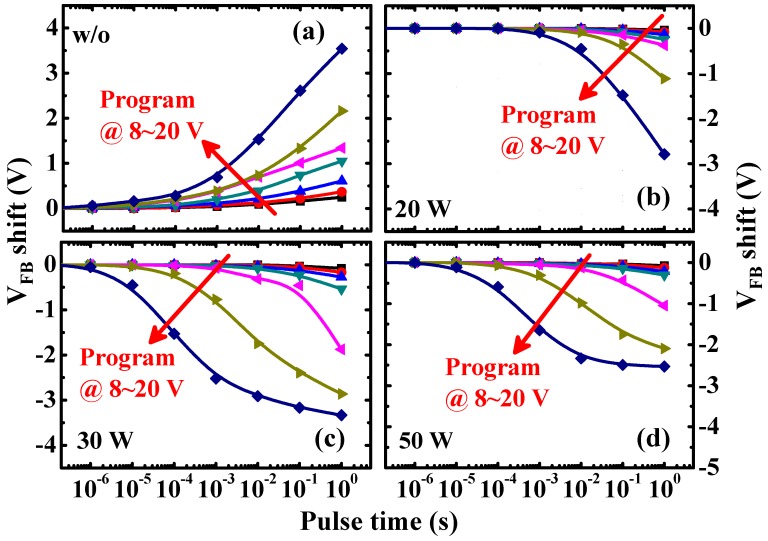
Programming characteristics of (**a**) w/o; (**b**) 20 W; (**c**) 30 W; and (**d**) 50 W samples with different pulse widths of *V*_g_ − *V*_FB_ of 8, 10, 12, 14, 16, 18, and 20 V. The pulse widths were in the range of 1 μs to 1 s. The *V*_FB_ shift towards the positive and negative directions was observed for the Au-NP NVMs without and with low-damage CF_4_ plasma treatment on the BO layer, respectively.

For the CF_4_-plasma-treated Au-NP NVMs, the flat-band voltage (*V*_FB_) might be modified and can be calculated using the *C*-*V* curves, as shown in Figure S3 of the Supplementary Materials. The *V*_FB_ values of the samples with the low-damage CF_4_ plasma treatment on the BO layer are roughly −1 V, which is larger than that obtained from the w/o sample. The *V*_FB_ shift can be ascribed to the passivation of the defects within the PECVD oxide layer by the CF_4_ plasma treatment [34]. In addition, compared to the w/o sample, the capacitances of the Au-NP NVMs with the low-damage CF_4_ plasma treatment on the BO layer are lower, leading to a lower dielectric constant of the fluorine-doped SiO_2_ layer, as proposed by T. Usami et al. [35]. For the Au-NP NVMs after programming, the shift of the *C*-*V* curves towards the positive and negative directions indicates that electrons and holes, respectively, are stored in Au-NPs [36]. The *C*-*V* curves of devices with different pulse widths of the programming voltage (*V*_g_ − *V*_FB_) of 20 V are shown in Figure S4 of the Supplementary Materials. The *V*_FB_ shifts of the devices with *V*_g_ − *V*_FB_ of 8 to 20 V were extracted from the *C*-*V* curves, and are presented in Figure 3. In Figure 3a, we can observe that the *V*_FB_ shifts towards the positive direction for the w/o sample, due to the electron injection from the *n*-type Si substrate into the Au-NPs. On the other hand, for the CF_4_-plasma-treated Au-NP NVMs, negative *V*_FB_ shift is achieved (Figure 3b), implying the hole injection from the Al gate into the Au-NPs. With the increase in the programming pulse biases and widths, the shift of the *V*_FB_ is larger. Moreover, for the devices with a larger plasma treatment power of 30 W, a more negative *V*_FB_ shift is obtained (Figure 3c). This is because more holes are injected and stored in the Au-NPs. However, if the CF_4_ plasma reactive power is more than 50 W, a decrease in *V*_FB_ shift is observed (Figure 3d), which will be discussed later. Furthermore, the erasing characteristics of Au-NP NVMs with and without low-damage CF_4_ plasma treatment on the BO layer were also investigated, as shown in Figure S5 of the Supplementary Materials.

### 3.3. Carrier Injection Mechanisms

**Figure 4:**
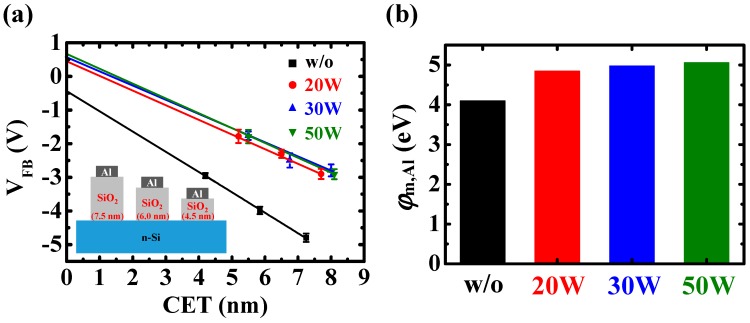
(**a**) Capacitance equilibrium thickness (CET) versus flat-band voltage characteristics of the Al/SiO_2_/*n*-Si structures with and without low-damage CF_4_ plasma treatment on SiO_2_ layers. The schematic Al/SiO_2_/*n*-Si structures of different SiO_2_ film thicknesses were shown in the inset figure. The extracted effective work functions of the Al gate (*φ*_m,Al_) with and without low-damage CF_4_ plasma treatment on SiO_2_ layers were shown in (**b**).

To realize the carrier injection mechanisms of Au-NP NVMs with and without the low-damage CF_4_ plasma treatment on the BO layer, the effective work function of the Al gate was extracted using Equation (2) [37]:

(2) $$V_{\mathrm{FB}}=\frac{\left(\varphi_{m,\mathrm{Al}}-\varphi_{n\mathrm{-Si}}\right)}{q}+\frac{Q_{\mathrm{ox}}}{A\varepsilon_{\mathrm{ox}}}\times t_{\mathrm{ox}}$$

where *φ*_m,Al_ is the effective work function of the Al gate with the low-damage CF_4_ plasma treatment on SiO_2_ layer, *φ_n_*_-Si_ is the *n*-type silicon work function of 4.43 eV, *Q*_ox_ is the oxide charges of the SiO_2_ layer, *t*_ox_ is the thickness of the SiO_2_ layer, *A* is the area of the devices, *ε*_ox_ is the dielectric constant of the SiO_2_ layer, and *q* is the electron charge of 1.6 × 10^−19^ C. Figure 4a shows the *V*_FB_ versus capacitance equilibrium thickness (CET) characteristics of the Al/SiO_2_/*n*-Si devices with the low-damage CF_4_ plasma treatment on SiO_2_ layer. The SiO_2_ films with thicknesses of 4.5, 6.0, and 7.5 nm were deposited by the PECVD system. After the device fabrication, the CETs of the SiO_2_ layers with and without the low-damage CF_4_ plasma treatment were obtained from the *C*-*V* curves, as shown in Figure S6 of the Supplementary Materials. By extrapolating the curves to the *y*-axis, the effective work functions of the Al gate on the low-damage CF_4_-plasma-treated SiO_2_ layer were extracted, as shown in Figure 4b. For the device without treatment, the effective work function of the Al gate is 4.1 eV, which is the same as that proposed by M. K. Achuthan and K. N. Bhat [38]. In addition, the effective work function of the Al gate on the low-damage CF_4_-plasma-treated SiO_2_ layer increases significantly to more than 5.0 eV, caused by the metal-induced gap states (MIGS) of the Al gate on the fluorinated SiO_2_ layer because of the large electronegativity of fluorine atoms [39].

**Figure 5:**
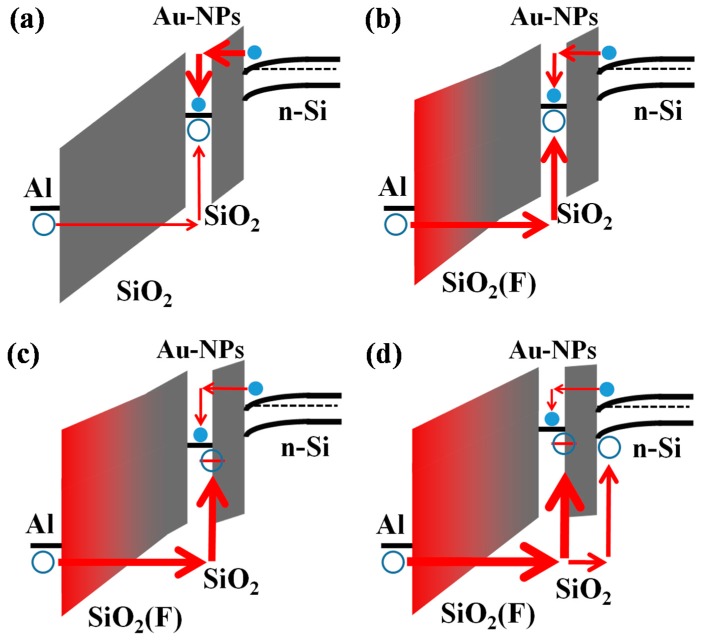
Schematic energy band diagrams of (**a**) w/o; (**b**) 20 W; (**c**) 30 W; and (**d**) 50 W samples at programming. Electrons and holes were injected from *n*-type Si substrate and Al gate, respectively, for the Au-NP NVMs without and with low-damage CF_4_ plasma treatment on the BO layer.

As a result of the large effective work function of the Al gate, the carrier injection mechanisms of the CF_4_-plasma-treated Au-NP NVMs are changed, as illustrated in the schematic energy band diagrams of Figure 5. For the w/o sample (Figure 5a), the substrate injection of electrons dominates at positive gate bias, leading to the positive *V*_FB_ shift shown in Figure 3a. In contrast, the gate injection of holes is dominant for the devices with the low-damage CF_4_ plasma treatment on the BO layer (Figure 5b–d), contributing to the negative *V*_FB_ shift shown in Figure 3b–d. According to Gauss’ Law, the displacement field (*D*) must be continuous at the TO and BO interface [40]. Thus, the lower dielectric constant of the CF_4_-plasma-treated BO layer results in a higher electric field on the gate bias. Because fluorine atoms are incorporated more and diffused deeper into the BO layer with the increase of the CF_4_ plasma reactive power, the electric field strength in the BO layer increases. Therefore, in Au-NP NVMs with a higher reactive power of CF_4_ plasma treatment, some holes are trapped at the interface between the Au-NPs and the TO layer [41], as shown in Figure 5c. Yeo et al. proposed the high MIGS at the Au and SiO_2_ interface owing to the dipole formation [42], which can be used for charge storage. If the CF_4_ plasma reactive power increases to more than 50 W, the electric field in the BO layer will be high enough to generate some hot holes that can potentially pass through the TO layer (Figure 5d), contributing to the decrease in *V*_FB_ shift shown in Figure 3d. Previously, we have proposed that the hole injection from the gate into the Au-NPs can be obtained by using the thick, stacked HfO_2_/Gd_2_O_3_/SiO_2_ oxide layers to prevent the injection of electrons from the substrate under the positive gate bias [41]. However, the data retention characteristics of the memory with Au and Gd_2_O_3_ bi-nanocrystals (BNCs) were not good enough (~30% at 10^4^ s) because of the serious interaction between the electrons and holes stored at the Gd_2_O_3_-nanocrystals (Gd_2_O_3_-NCs) and Au-NPs, respectively.

### 3.4. Data Retention Behaviors

**Figure 6:**
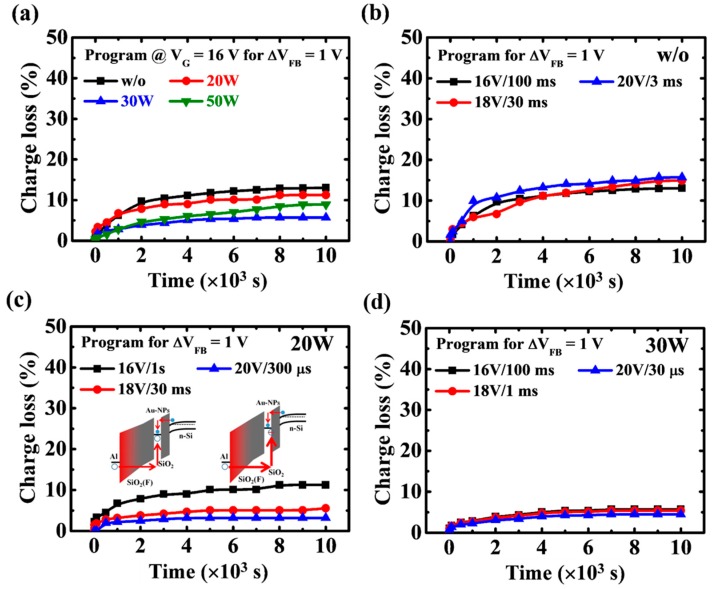
(**a**) Data retention characteristics of the Au-NP NVMs with low-damage CF_4_ plasma treatment on the BO layer. The data retention properties at different programming voltages of the w/o, 20 W, and 30 W samples are shown in (**b**–**d**), respectively. Schematic energy band diagrams of the 20 W sample at low and high programming voltages were illustrated in the inset figure of (**c**).

Figure 6a shows the data retention characteristics of the Au-NP NVMs with the low-damage CF_4_ plasma treatment on the BO layer. All samples were first programmed at 16 V to achieve a flat-band voltage shift (Δ*V*_FB_) of 1 V. The charge loss is defined by the formula [9]

(3) $$\mathrm{Charge}\;\mathrm{loss}\;(\%)=\left(\frac{V_{p}-V_{r}}{V_{p}-V_{0}}\right)\times 100\%$$

where *V*_0_ is the *V*_FB_ of the fresh sample, *V*_p_ is the *V*_FB_ of the sample being programmed, and *V*_r_ is the *V*_FB_ of the programmed sample measured after a certain retention time. In this figure, lower charge loss is achieved in Au-NP NVMs with CF_4_ plasma treatment on the BO layer compared to those without treatment. The reduced charge loss can be explained by the holes with high effective mass stored in Au-NPs. With the increase of the CF_4_ plasma reactive power, the charge loss can be further reduced to lower than 5% at 10^4^ s, which is ascribed to the holes trapped at the interface between the Au-NPs and the TO layer, as shown in Figure 5c. However, for the 50 W sample, an increase in charge loss is observed, for which the plasma damage induced by the high reactive power is responsible. To deeply understand the mechanism of the low charge loss of the CF_4_-plasma-treated Au-NP NVMs, the data retention properties are evaluated at different programming voltages of the w/o, 20 W, and 30 W samples, as shown in Figure 6b–d, respectively. The *C*-*V* curves of the w/o and 20 W samples at low and high programming voltages under data retention measurement are displayed in Figure S7 of the Supplementary Materials. It can be observed that for the w/o sample, the charge loss is still high, even at the programming voltage of 20 V, whereas for the 20 W sample, the charge loss is significantly reduced with the increase in programming voltage due to the holes trapped at the interface between the Au-NPs and the TO layer by the high electric field [41], as illustrated in the schematic energy band diagrams of the inset figures of Figure 6c. For the 30 W sample, because the holes are already trapped at the interface between the Au-NPs and the TO layer by the low electric field (Figure 5c), the charge loss remains low at high programming voltages, as shown in Figure 6d. 

**Figure 7:**
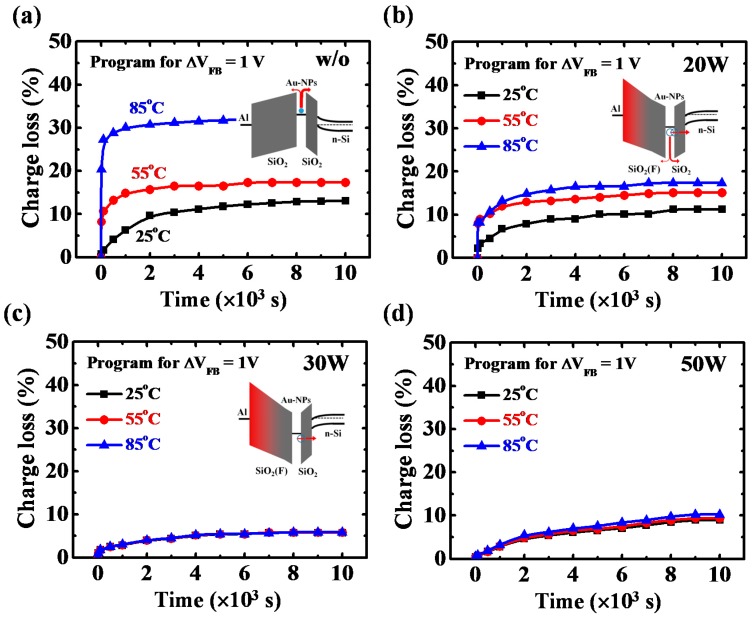
(**a**) Data retention characteristics of (**a**) w/o; (**b**) 20 W; (**c**) 30 W; and (**d**) 50 W samples at elevated measurement temperatures. The measurement temperatures were in the range of 25 to 85 °C. Schematic energy band diagrams of the w/o, 20 W and 30 W samples at retention state were illustrated in the inset figure of (**a**–**c**), respectively.

The charge storage mechanisms of the Au-NP NVMs with and without the low-damage CF_4_ plasma treatment on the BO layer can be further confirmed by the temperature dependence of the charge loss behaviors, as shown in Figure 7. The w/o sample exhibits an obvious increase in charge loss when the measurement temperature increases from 25 to 85 °C (Figure 7a), implying that the electrons are stored in the Au-NPs. The thermionic emission of electrons in Au-NPs contributes to the significant dependence of temperature on charge loss. Furthermore, for the 20 W sample, a slight increase in charge loss is observed (Figure 7b), because the holes are stored in both the Au-NPs and at the interface between the Au-NPs and the TO layer. The holes trapped at the interface between the Au-NPs and the TO layer will lose through the TO layer by the tunneling leakage, reducing the dependence of temperature on charge loss. Conversely, a nearly negligible increase in charge loss is obtained for the 30 W and 50 W samples (Figure 7c,d), indicating that the holes are stored mainly at the interface between the Au-NPs and the TO layer.

## 4. Conclusions

In summary, the gate-injected Au-NP NVMs with low-damage CF_4_ plasma treatment on the BO layer were proposed, which exhibit superior data retention properties. To examine the holes injected from the Al gate of the CF_4_-plasma-treated memory devices, some material analysis techniques such as SIMS, XPS, UPS, SEM, and HRTEM were performed. With the increase in the CF_4_ plasma reactive power, more fluorine atoms were incorporated in the BO layer, leading to a higher electric field and Fermi-level pinning of the Al gate towards the valence band edge of BO layer. Thus, more holes were injected from the gate and trapped at the interface between the Au-NPs and the TO layer, contributing to the superior data retention characteristics of the CF_4_-plasma-treated Au-NP NVMs for use in highly reliable consumer electronics.

## Supplementary Materials

The following are available online at http://www.mdpi.com/2079-4991/7/11/385/s1: Figure S1: HRTEM image of Au-NP NVMs; Figure S2: SEM image of Au-NPs on SiO_2_ tunneling oxide layer; Figure S3: capacitance versus voltage (*C*-*V*) characteristics of Au-NP NVMs with low-damage CF_4_ plasma treatment on BO layer; Figure S4: *C*-*V* curves of (a) w/o, (b) 20 W, (c) 30 W, and (d) 50 W samples at the programming voltage (*V*_g_ − *V*_FB_) of 20 V; Figure S5: erasing characteristics of (a) w/o and (b) 20 W samples at the erasing voltages of −12 to −18 V and −14 to −26 V, respectively; Figure S6: *C*-*V* curves of different thicknesses of SiO_2_ films (a) without and with low-damage CF_4_ plasma treatment of (b) 20 W, (c) 30 W, and (d) 50 W; Figure S7: *C*-*V* characteristics of the w/o sample programmed at (a) 16 and (b) 20 V, respectively, and the 20 W sample programmed at (c) 16 and (d) 20 V, respectively, at the retention time of 10^4^ s.
