# Supplementary material for: Data Retention Characterization of Gate-Injected Gold-Nanoparticle Non-Volatile Memory with Low-Damage CF4-Plasma-Treated Blocking Oxide Layer
Source: Nanomaterials (Basel). 2017 Nov 10;7(11):385. doi: 10.3390/nano7110385 (PMC5707602; doi:10.3390/nano7110385)
Supplement: Supplementary file 1 [file nanomaterials-07-00385-s001.pdf]

Supplementary Materials for

Data Retention Characterization of Gate-Injected Gold-Nanoparticle Non-Volatile Memory with Low-Damage CF_4 Plasma Treated Blocking Oxide Layer

Yu-Hua Liu [1,†], Chyuan-Haur Kao [1,2,3,†], Tsung-Chin Cheng [4], Chih-I Wu [4,5,6] and Jer-Chyi Wang [1,2,7,*]

[1] Department of Electronic Engineering, Chang Gung University, Guishan Dist. 33302, Taoyuan, Taiwan
[2] Department of Electronic Engineering, Ming Chi University of Technology, Taishan Dist. 24301, New Taipei City, Taiwan
[3] Kidney Research Center, Department of Nephrology, Chang Gung Memorial Hospital, Linkou, Guishan Dist. 33305, Taoyuan, Taiwan
[4] Graduated Institute of Photonics and Optoelectronics, National Taiwan University, Taipei 10617, Taiwan
[5] Department of Electrical Engineering, National Taiwan University, Taipei 10617, Taiwan
[6] Electronic and Optoelectronic System Research Laboratories, Industrial Technology Research Institute, Chutung 31057, Hsinchu, Taiwan
[7] Department of Neurosurgery, Chang Gung Memorial Hospital, Linkou, Guishan Dist. 33305, Taoyuan, Taiwan

†These authors contributed equally to this work
*Corresponding authors, e-mail: jcwang@mail.cgu.edu.tw

Contents:

S1. HRTEM Image of Au-NP NVMs 2

S2. SEM Image of Au-NPs on SiO_2 Tunneling Oxide Layer 3

S3. Capacitance *versus* Voltage (*C-V*) Characteristics of Au-NP NVMs with Low-Damage CF_4 Plasma Treatment on BO Layer 4

S4. *C-V* Curves of Low-Damage CF_4-Plasma-Treated Au-NP NVMs Under Programming 5

S5. Erasing Characteristics of Low-Damage CF_4-Plasma-Treated Au-NP NVMs 6

S6. *C-V* Curves of Different Thicknesses of SiO_2 Films with Low-Damage CF_4 Plasma Treatment 7

S7. *C-V* Characteristics of Programmed Au-NP NVMs at Retention State 8

S1. HRTEM Image of Au-NP NVMs

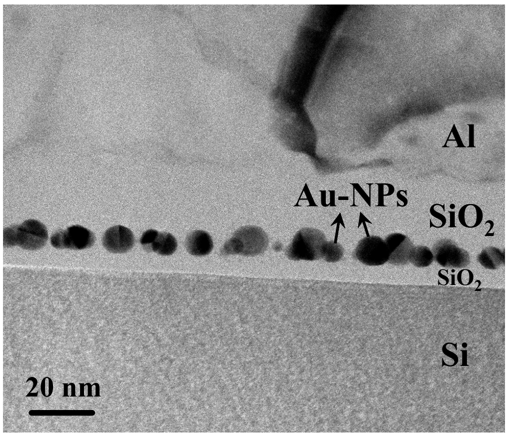

Figure S1. HRTEM image of the Au-NP NVMs. Distinct Au-NPs were observed on the SiO_2 tunneling oxide layer. The image also confirmed the film thicknesses of the TO and BO layers, for the further study of carrier injection.

S2. SEM Image of Au-NPs on SiO_2 Tunneling Oxide Layer

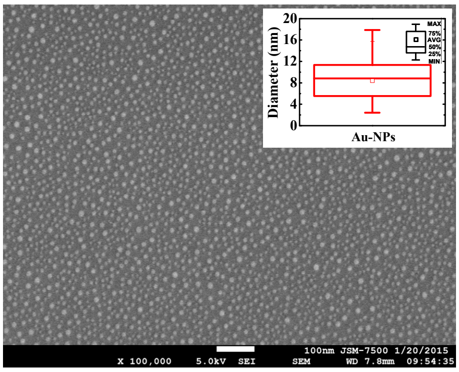

Figure S2. SEM image of Au-NPs on SiO_2 tunneling oxide layer. First, a 2-nm-thick Au film was deposited by a thermal evaporator at 10^{-6} Torr with a pure Au bullet (99.99% purity). Subsequently, all samples were subjected to the rapid thermal annealing (RTA) system at 700 ^{o}C for 30 s to form the Au-NPs. To determine the Au-NP dot size, the software of ImageJ was used to analyze the SEM image. The statistical distribution of the Au-NP size was presented in inset of this figure and the average particle size was found to be 8.4 nm in diameter, which was almost the same as that obtained in HRTEM image. In addition, the corresponding standard deviation extracted from the image was 3.85 nm and the dot density was calculated to be 1.2×10^{12} cm^{-2}.

S3. Capacitance *versus* Voltage (*C-V*) Characteristics of Au-NP NVMs with Low-Damage CF_4 Plasma Treatment on BO Layer

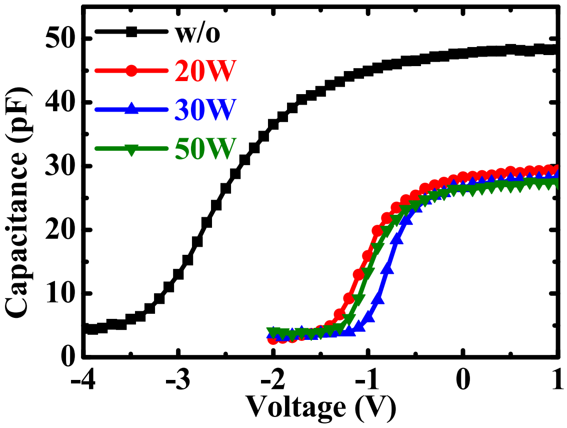

Figure S3. Capacitance versus voltage (*C-V*) characteristics of Au-NP NVMs with low-damage CF_4 plasma treatment on BO layer. The *C-V* curves were used to calculate the flat-band voltage (V_{FB}) of the samples with the low-damage CF_4 plasma treatment on the BO layer. The V_{FB} values of the samples with the low-damage CF_4 plasma treatment on the BO layer are roughly -1 V, which is larger than that obtained from the w/o sample.

S4. *C*-*V* Curves of Low-Damage CF_4-Plasma-Treated Au-NP NVMs Under Programming

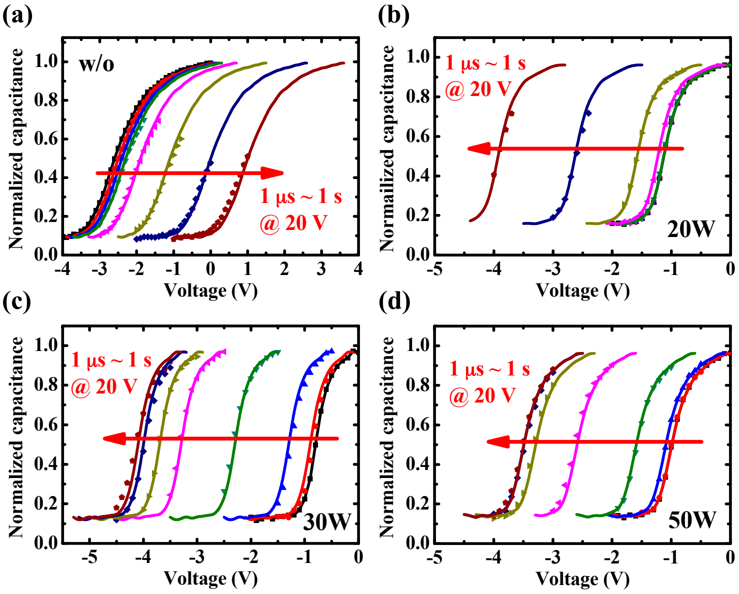

Figure S4. *C*-*V* curves of (a) w/o, (b) 20W, (c) 30W, and (d) 50W samples at the programming voltage (V_g-V_{FB}) of 20 V. The pulse widths were in the range of 1 μs to 1 s. The curves shift toward positive and negative directions indicate the injection of electrons from n-type Si substrate into Au-NPs and the injection of holes from Al gate into Au-NPs, respectively.

S5. Erasing Characteristics of Low-Damage CF_4-Plasma-Treated Au-NP NVMs

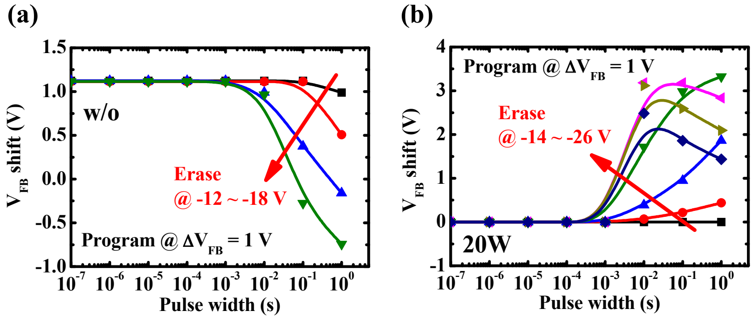

Figure S5. Erasing characteristics of (a) w/o and (b) 20W samples at the erasing voltages of -12 to -18 V and -14 to -26 V, respectively. All samples were first programmed to obtain a ΔV_{FB} of 1 V. The change of V_{FB} indicates the erase of electrons and holes from Au-NPs of the w/o and 20W samples, respectively.

S6. *C-V* Curves of Different Thicknesses of SiO_2 Films with Low-Damage CF_4 Plasma Treatment

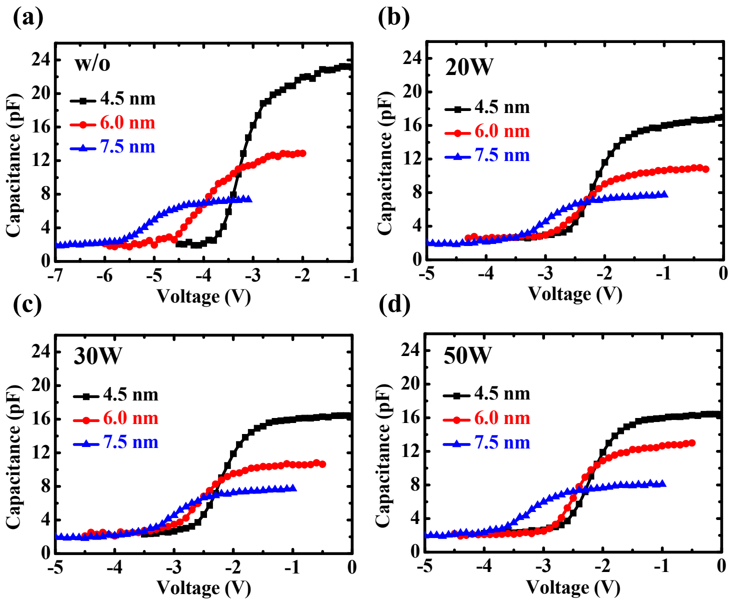

Figure S6. *C-V* curves of different thicknesses of SiO_2 films (a) without and with low-damage CF_4 plasma treatment of (b) 20 W, (c) 30 W, and (d) 50 W. The SiO_2 films with the thicknesses of 4.5, 6.0, and 7.5 nm were deposited by the PECVD system. These curves were used to extract the effective work-function of Al gate on the CF_4-plasma-treated SiO_2 films.

S7. *C-V* Characteristics of Programmed Au-NP NVMs at Retention State

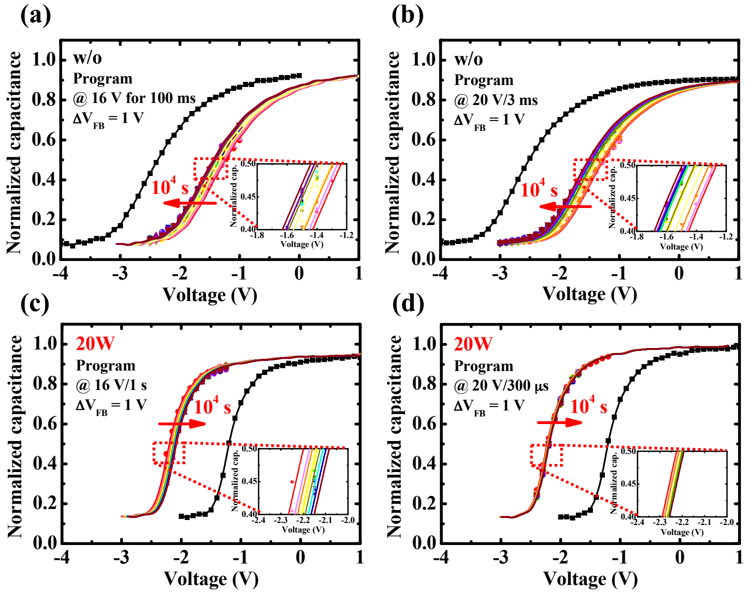

Figure S7. *C-V* characteristics of the w/o sample programmed at (a) 16 and (b) 20 V respectively and the 20W sample programmed at (c) 16 and (d) 20 V respectively at the retention time of 10^4 s. All samples were first programmed to achieve a ΔV_{FB} of 1 V. The enlarged curves of each of the samples were shown in the inset figure. The *C-V* shifts indicate the higher gate bias will improve the data retention characteristics of the 20W sample.
